# Oral Mycobiota: A Narrative Review

**DOI:** 10.3390/dj12040115

**Published:** 2024-04-19

**Authors:** Carmen Liliana Defta, Cristina-Crenguţa Albu, Ştefan-Dimitrie Albu, Claudia Florina Bogdan-Andreescu

**Affiliations:** 1 Department of Microbiology, Faculty of Dentistry, “Carol Davila” University of Medicine and Pharmacy, 020021 Bucharest, Romania; carmen.defta@umfcd.ro; 2 Department of Genetics, Faculty of Dentistry, “Carol Davila” University of Medicine and Pharmacy, 020021 Bucharest, Romania; 3 Department of Periodontology, Faculty of Dentistry, “Carol Davila” University of Medicine and Pharmacy, 020021 Bucharest, Romania; stefan-dimitrie.albu@drd.umfcd.ro; 4 Department of Speciality Disciplines, Faculty of Dental Medicine, “Titu Maiorescu” University, 031593 Bucharest, Romania; claudia.andreescu@prof.utm.ro

**Keywords:** oral microbiota, mycobiota, dysbiosis, next-generation sequencing

## Abstract

Numerous studies have proven the important role of the oral microbiota in health and disease. The dysfunctionality of the oral microbiota, known as dysbiosis, is incriminated in dental caries, periodontal disease, oral infectious diseases, oral cancer, and systemic disease. The lesser-known component of the oral microbiota, the mycobiota, is now assiduously investigated. Recent technological developments have helped foster the identification of new fungal species based on genomic research. Next-generation sequencing has expanded our knowledge about the diversity, architecture, and relationships of oral microorganisms within the oral cavity. The mycobiome structure and relationships with the bacteriome have been studied to identify a mycobiotic signature. This review aimed to emphasize the latest knowledge of the oral mycobiome.

## 1. Introduction

In recent years, the awareness of the oral microbiome and its relationship to the state of health or disease has undergone a tremendous evolution, mainly due to the development of microbe identification technology [1].

The oral cavity shelters a complex community of microbes: bacteria, fungi, viruses, protozoa, and archaea [2]. The resident microorganisms are known as commensal or normal microbiota and are beneficial for the host [3]. They restrain the spreading of opportunistic pathogens and regulate the inflammatory responses of the host [4]. The commensal oral microbiota is crucial to the health of the host because it is involved in oral health, oral pathology, and systemic diseases [5,6].

“Oral microbiome”, “oral microflora”, or “oral microbiota” are the terms most commonly used to describe the microbial community residing in the human oral cavity [5].

The oral microbiota consists of three major components: the oral bacteriome (bacterial), the oral mycobiome (fungal), and the oral virome or oral virobiome (viral) [7,8].

Of these, bacteria are the most studied microbial agents of the oral microbiota, whereas the viral component of microbiota, or oral virome, and the fungal component of microbiota, or mycobiome, are less investigated and were considered minor components of oral microbiota [7,8,9]. However, in recent years, the number of papers which investigated mycobiota has increased, indicating an expansion in this field.

Commensal fungi are important components for the health and disease of the host; they can have profound effects on the immune system. The disturbance of the oral mycobiota has been suggested to influence the evolution of infectious diseases, especially among immunocompromised patients, like HIV-infected patients or cancer patients.

Significant evidence demonstrates that numerous diseases are strongly related to gut mycobiota [10,11,12]. In addition, recent studies have established a relationship between the oral mycobiome and pancreatic adenocarcinoma [13] or fatty liver disease [14].

The oral mycobiota is the most diverse and complex mycobiome in healthy individuals [15], but the components of the oral mycobiome are not yet completely known. 

The main component of commensal mycobiota is *Candida*. However, there are many other fungal species within the oral mycobiome. Many fungal species are unculturable in conventional laboratory media [16], which is the main limitation in the identification of the true complexity of the fungal microbiome. Nonetheless, the apparition of new technologies based on DNA extraction from saliva, like universal primers and amplicon sequencing, circumvent the limitations of cultivation approaches [17].

Nowadays, next-generation sequencing (NGS) may fill in the gap regarding the identification of the fungal genera [18,19]. The expansion of the NGS techniques has supported the investigation of the fungal microbiome, which helps to clarify the mycobiome’s role. The ability of fungal species to prosper in extreme environmental conditions and produce secondary metabolite components with bioactivities varying from possible therapeutics to toxins is now being investigated [20].

The aim of this narrative review is to identify an oral mycotic signature that could be linked to specific oral or systemic disorders.

Consequently, to fulfill the purpose of our study, we searched and selected from the Internet (PubMed and Google Scholar) the most recent publications in the field from the last five years that studied oral mycobiome. The keywords were oral and mycobioma. The exclusion criteria were animal mycobiome studies, other human mycobiome studies, except oral, and oral medicine studies.

## 2. Commensal Mycobiome

There are several studies which try to define the commensal mycobiome in healthy individuals. Furthermore, the term core or basal mycobiome is frequently used to describe the mycobiome’s components that remain relatively constant across time in healthy individuals.

A study from 2010 described the oral mycobiome of healthy individuals and identified the following genera: *Candida*, *Cladosporium*, *Aureobasidium*, *Saccharomycetales*, *Aspergillus*, *Fusarium*, and *Cryptococcus* [21]. The researchers isolated a number of 9 to 23 fungal species from the oral cavity of each individual with the pan-fungal internal transcribed spacer primers [21]. This study illustrated the variety of oral fungal microbiota by discovering 85 genera, including 74 culturable and 11 non-culturable [21].

Ghannoum and colleagues described thirteen elements in the core mycobiome: *Alternaria*, *Aspergillus*, *Aureobasidium*, *Candida*, *Cladosporium*, *Cryptococcus*, *Dothioraceae*, *Eurotium*, *Fusarium*, *Glomus*, *Saccharomyces*, *Saccharomycetales*, and *Teratosphaeria* [21]. The following species were identified: *Alternaria tenuissima*, Alternaria triticina, Aspergillus amstelodami, Aspergillus caesiellus, Aspergillus flavus, Aspergillus oryzae, Aspergillus penicillioides, Aspergillusruber, Candida albicans, Candida khmerensis, Candida metapsilosis *Candida parapsilosis*, *Candida tropicalis*, Cladosporium cladosporioides, Cladosporium herbarum, and Cladosporium *sphaerospermum.*

Another study from 2014 discovered the genus *Malassezia* as an important commensal in oral mycobiome, using high throughput sequencing of internal transcribed spacer amplicons from salivary samples [22]. Dupuy and colleagues identified seven of above-mentioned genera in more than half of the subjects (*Candida*, *Cladosporium*, *Alternaria*, *Aspergillus*, *Fusarium*, *Cryptococcus*, and *Aureobasidium*) [22]. Five genera were recognized in high frequency in this research, but they were not components of the core oral mycobiome proposed by Ghannoum et al.: *Malassezia*, *Irpex*, *Cytospora/Valsa*, *Lenzites/Trametes*, and *Sporobolomyces/Sporidiobolus.*

A study of fungal species in oral samples of healthy young adults identified *Candida*, *Rhodotorula*, *Penicillium*, *Aspergillus*, *Cladosporium*, *Trichoderma*, *Scedosporium*, *Alternaria*, and *Rhizopus* as more prevalent fungi [23]. *Candida albicans*, Candida parapsilosis, and *Candida tropicalis* were detected among Candida carriers [23]. *Aspergillus fumigatus* was prevalent, and *Aspergillus flavus* and Aspergillus glaucus were rare [23]. The stability of oral mycobiome was observed at 15 days and six months later, and a great interindividual variability with consistent intraindividual stability was noticed over time [23].

An investigation of oral commensal flora in the Chinese healthy population revealed that the genus *Candida* is the prominent genus of healthy oral mycobiota [24]. *Candida* is followed by *Fusarium*, *Rhodotorula*, *Alternaria*, and *Cladosporium* [24].

The identified components of the oral mycobiome are listed in Table 1.

Few studies have tried to elucidate the fundamental mycobiome composition. All studies were based on DNA extraction from saliva samples. The information provided outlines some important points about the study of the oral mycobiome. The findings suggest a few key insights into the composition and diversity of the fungal communities residing in the human mouth.

We could distinguish two common genera, *Candida* and *Cladosporium*, identified across all studies. This underlines their significant presence within the oral ecosystem. Candida, especially, is known for its commensal behavior in human hosts, although certain species, like *Candida albicans*, can become opportunistic pathogens under specific conditions. The presence of Cladosporium, a common environmental fungus, suggests either a non-pathogenic role within the mouth or frequent introduction from the environment.

The fact that genera such as *Alternaria*, *Aspergillus*, and *Fusarium* were found in the majority of studies (three out of four) highlights the diversity of the oral mycobiome. These genera are known for their wide distribution in the environment, and their presence in the oral cavity might reflect either transient colonization or a more stable association with the host.

The identification of *Aureobasidium*, *Cryptococcus*, and *Rhodotorula* in half of the conducted research indicates variability in mycobiome composition across different individuals or populations. This variability could be due to differences in geographic location, dietary habits, oral hygiene, or methodological aspects like DNA extraction and sequencing techniques.

The mention of other genera being reported in singular studies suggests that there’s still much to learn about the oral mycobiome’s diversity. These less commonly reported fungi might represent transient members of the mycobiome, species with a niche role in the oral ecosystem, or simply variability in detection and identification methods.

Based on the mentioned studies, the core mycobiome is characterized by the presence of multiple fungal genera, with Candida as the most predominant.

## 3. Caries and Oral Mycobiome

The role of fungi in the etiology and evolution of dental caries has been contradictory. The studies are related to *Candida albicans*, the most prominent component of oral fungal biota, and are focused on child caries [25].

Clinical studies about early childhood caries, like that of Neves et al. and Peretz et al., revealed the absence of an association between caries risk and *Candida albicans* existence or no significant difference between caries status and *Candida albicans* prevalence [25,26].

On the contrary, research performed by Thomas et al., carried out on a larger group of very young children, revealed a substantial increase in *Candida albicans* count in severely early childhood caries children in comparison with caries-free children [27]. The authors suggested that *Candida albicans* may be responsible for the initiation and development of severe early childhood caries in very young children due to immature immune systems.

The association between caries and *Candida albicans* was confirmed by researchers using a mice caries model. A study revealed that *Candida albicans* could produce caries when mice were fed a high-sugar diet [28]. Another study proved the important role of *Candida albicans* in the development of rat root caries [29]. *Candida albicans* induce bacteriome dysbiosis with an increased concentration of *Streptococcus mutans* [29].

Other reports have discovered that *Candida albicans* is frequently identified in association with *Streptococcus mutans*, wellknown as caries pathogen, in plaque samples from children with early childhood caries [30,31,32]. These findings suggest that the interaction between oral bacteria and fungi may have a significant role in caries development.

Based on these discoveries, O’Connell and colleagues attempted to clarify the complex microbial interactions involved in caries etiology and progression [33]. They aimed to characterize the mycobiome resident in supragingival dental plaque during the different stages of caries evolution [33]. The taxonomic profiles of the microbiome from supragingival dental plaque samples were created with the internal transcribed spacer amplicon sequencing for children with different caries status: children caries free, children with enamel active caries, and children with dentin active caries [33].

In this study, a number of 139 fungal species were recognized [33]. The most abundant species was *Candida albicans*, followed by *Candida dubliniensis*. The researchers revealed a significant difference between the supragingival plaque of caries-free children and the supragingival plaque of children with dentine caries. *Candida albicans*, *Candida dubliniensis*, *Nigrospora oryzae*, and an unclassified *Microdochium* species were associated with caries, while 12 other taxa were connected with health. *Candida dubliniensis* increased progressively as caries reached dentine. In contrast, four health-associated fungal taxa (*Debaryomyces*, *Rhodotorula*, *Aureobasidium*, and Aspergillus) can antagonize the cariogenic *Streptococcus mutans* via xylitol production [33].

The role of *Candida* in the etiology and progression of dental caries has not been established clearly. It seems that there is more of a microbial interaction between fungi and bacteria than the virulence of certain species in the etiopathogeny of caries. *Candida albicans* possess important abilities for caries development: they can develop thick biofilms, ferment dietary sugars, and produce enzymes that break collagen [34].

## 4. Periodontitis and Oral Mycobiome

A research investigation explored the link between periodontitis and the oral mycobiome [35]. The study utilized a pan-fungal internal transcribed spacer to identify genes in the DNA extracted from the saliva of two distinct groups, individuals with periodontal disease and subjects with good oral health [35].

The authors identified at least 81 genera and 154 fungal species across all samples. The most frequently detected genera are *Candida* and *Aspergillus* (isolated from all participants), followed by *Penicillium*, *Schizophyllum*, *Rhodotorula*, and *Gibberella* [35]. There were no significant differences in the diversity or composition of the oral mycobiome between individuals with periodontal disease and those without it [35]. Although the genus *Candida* was previously associated with periodontal disease in culture-based studies [36,37,38], in this study, *Candida* was higher in individuals with periodontal disease, but this difference was not statistically significant [37]. However, within the group of individuals with periodontal disease, the amount of *Candida* increased as the number of missing teeth increased [35].

These results are in accordance with another study, which identified *Candida albicans*, *Candida glabrata*, and *Candida dubliniensis* in saliva samples from community-dwelling elderly, predominant among denture wearers [39]. According to the findings, these species tend to prosper when the immune system of the host weakens [39]. Indeed, *Candida dubliniensis* is frequently found in individuals with a compromised immune response [40,41] and is associated with tobacco smoking [42,43].

In the era of infectogenomics, a better understanding of the host genetic variant that predisposes to both subgingival microbial colonization and the progression of periodontal disease could contribute to a better understanding of the pathogenesis and management of periodontal disease [44,45].

The definitive role of fungi in the onset and advancement of periodontitis remains to be firmly established. The principal fungal genera isolated from the oral mycobiome of individuals with periodontitis align with those identified within the core mycobiome.

## 5. Peri-Implantitis and Oral Mycobiome

Recent research has focused on the association between peri-implantitis and mycobiome since peri-implant diseases are related to the oral microbiome. The most prominent oral fungus, *Candida albicans*, seems to play a significant role in the development of peri-implant pockets due to the development of a biofilm on titanium surfaces, which permits the growth of pathogenic bacteria [46].

A systematic review of *Candida*’s role in peri-implantitis concluded that *Candida* is an important part of the microbiota within peri-implantar sulcus [47]. The most isolated species are *Candida albicans*, *Candida parapsilosis*, *Candida tropicalis*, and *Candida dubliniensis* [47].

A pilot study relates *Aspergillus restrictus* and *Candida parapsilosis* to subjects with peri-implantitis when compared to healthy controls with the aim of establishing the role of mycobiome in peri-implantitis [48]. In this study, *Porphyromonas gingivalis* is the bacteria species more abundant in subjects with peri-implantitis [47], a common pathogenic bacteria associated with peri-implantitis [49]. It is possible that the association between *Porphyromonas gingivalis*, *Aspergillus restrictus,* and *Candida parapsilosis* increases the virulence of the peri-implantar microbiome. A previous study proved increased virulence of *Candida albicans* in association with certain bacterial species that contribute to peri-implantitis [50]. Therefore, the association between bacterial and fungal species in peri-implant sulcus is critical for the development of peri-implantitis.

The mycobiome is currently in the spotlight in biofilm-mediated oral diseases such as caries, periodontitis, and peri-implantitis, owing to its ability to form a thick biofilm, which harbors diverse bacterial species and promotes dysbiosis. *Candida albicans* has the ability to adhere to dental implants [51]. There is no proven mechanism for bone resorption produced by *Candida albicans*, but a possible model could be *Candida* colonization, production of biofilm, bacterial accumulation, and microbiological shift from a commensal to a pathologic profile.

## 6. Oral Fungal Infection and Oral Mycobiome

The most common cause of oral fungal infections is *Candida* species, but there are other fungal infections that can have oral manifestations, such as mucormycosis, aspergillosis, blastomycosis, histoplasmosis, cryptococcosis, and coccidioidomycosis [52].

Most solitary or primary oral and maxillofacial fungal infections are rare, except for oral candidiasis, which occurs in patients who are immunocompromised [52]. 

The increase in *Candida* is presumed to result from an overgrowth of indigenous species in a permissive host environment [53]. Over 17 different *Candida* species have been known to cause oral fungal infections. While *Candida albicans* is the most common pathogen of candidosis, other species such as *Candida glabrata*, *Candida parapsilosis*, *Candida tropicalis*, and *Candida krusei* have also been implicated in cases of oral or disseminated candidosis and candidemia [54]. 

An interesting study compared the oral mycobiome of subjects with candidiasis with the oral mycobiome of healthy individuals [55]. Imabayashi et al. discovered that the number of fungal species in healthy controls increased with aging, especially non-*Candida albicans* species [55]. Those with candidiasis had their mycobiomes dominated by *Candida albicans*, as expected [55]. However, *Candida tropicalis* and *Candida dublinensis* were also detected in some subjects with moderately high abundance [55]. This study proved that fungal diversity increases with advancing age and suggests that aging could be associated with the pathogenesis of oral candidiasis [55].

Significant evidence revealed a positive association between oral microbiome and advancing age [18,56,57]. Ikebe et al. discovered a positive association between oral mycobiota and advancing age, tooth loss, denture wearing, and low salivary flow rates [56]. The retention areas from removable dentures may be the main reason for these findings [56]. Also, the reduced salivary flow and the atrophic mucosa associated with advancing age [58] may have a significant role in shifting the composition of the oral microbiome.

Another study confirmed that oral *Candida* is associated with the aging process [58]. However, it is also associated with untreated decayed teeth, prosthetic teeth, and salivary pH levels [57]. This suggests that there is a permanent balance within the oral microbiome between the fungus and bacteria, which are harbored by healthy teeth and surrounding tissues [57]. Active caries and tooth loss can disrupt this balance [57].

Oral mycosis is predominantly associated with *Candida* species, particularly *Candida albicans*. Factors such as aging and wearing dentures are believed to favor conditions for the overgrowth of *Candida*.

## 7. Oral Potentially Malignant Disorders, Oral Cancer, and Oral Mycobiome

Oral potentially malignant disorders are defined as “any oral mucosal abnormality that is associated with a statistically increased risk of developing oral cancer” [59]. They included leukoplakia, erythroplakia, proliferative verrucous leukoplakia, oral lichen planus, oral submucous fibrosis, palatal lesions in reverse smokers, lupus erythematosus, epidermolysis bullosa, and dyskeratosis congenital [59].

The oral potentially malignant disorders could be discovered in different sites in the oral cavity and could be described as morphological changes plaque/plateau, smooth, grooved, wrinkled, granular, and atrophic with different colors, such as white, red, and mixed white-red [60]. 

The risk factors for oral potentially malignant disorders are tobacco and alcohol, human papilloma virus, areca nut chewing, and microbiome alterations, among others [61]. The process by which microbiome alteration generates modifications in the oral mucosa is related to the increase in pathogenic bacteria, which trigger cell proliferation and permanent genetic mutations by their products and metabolites [62,63]. 

The role of mycobiome in the etiopathology of oral potentially malignant disorders and oral cancer is not clearly identified, but fungi have been associated with these conditions. 

*Candida albicans* is detected among subjects with oral potentially malignant disorders and oral cancer [64]. 

It is common for patients with oral lichen planus to have oral *Candida* infections [65,66,67]. Non-erosive oral lichen planus is frequently associated with *Candida albicans*, while *Candida glabrata* and *Candida krusei* are more commonly found in patients with erosive oral lichen planus [67,68].

Oral swabs from patients with active oral lichen planus often revealed the presence of non-*Candida albicans* species, such as *Candida glabrata*, *Candida dublinensis*, *Candida krusei*, and *Candida parapsilosis* [41]. However, *Candida albicans* remains the most frequently isolated species [41]. These non-albicans species have been linked to patients who wear removable dentures [41].

Oral leukoplakia, particularly nonhomogeneous leukoplakia, has a higher risk of transformation into oral cancer [69]. Recent studies support the correlation between *Candida* and leukoplakia [70]. Gupta et al. conducted a study in which they cultured *Candida albicans* from saliva samples collected from individuals with leukoplakia and oral squamous cell carcinoma and compared them with those from the control group [70]. The growth of *Candida albicans* was only observed in severe dysplastic patients, while mild and moderate dysplasia showed no *Candidal* growth [70]. The genotype A of *Candida* was suggested to be associated with oral leukoplakia [71,72].

These findings are in accordance with previous studies, which relate *Candida* species and their biotypes with premalignant lesions, such as the oral lichen planus and leukoplakia [73,74], and also with oral carcinogenesis [74]. 

*Candida albicans* and *Candida tropicalis* were associated with mild/moderate dysplasia in a study that compared biopsy samples from non-smoker subjects with dysplasia, carcinoma in situ, oral squamous cell carcinoma, and histologically benign lesions [75].

The *Candida* species isolated from oral tongue squamous cell carcinoma patients was found to generate more virulence factors than the control group [76].

According to one study, *Candida parapsilosis* and *Candida albicans* genotype B were found to be more prevalent in non-oral cancer patients, while *Candida albicans* genotype A was more commonly detected in oral cancer patients [77]. The researchers also demonstrated that *Candida parapsilosis* and *Candida albicans* genotype B had greater virulence due to their higher ability to form biofilms, produce more carcinogenic acetaldehyde, and exhibit higher metabolic activity [78]. Additionally, *Candida albicans* were discovered to produce nitrosamines, which are potent carcinogens [79].

The dysbiosis of oral biota is positively related to the occurrence of tumor tissues in the oral cavity. An investigation of the bacteriome, mycobiome, and their interaction in associations with oral tongue cancer discovered that the fungal phylum *Glomeromycota* was significantly decreased in the tumor group compared to their matched control [80]. The researchers have proved that the bacterial diversity and richness, and the fungal richness, were significantly reduced in the tumor group [80,81]. Among the bacterial phyla, *Firmicutes* were the most abundant and significantly associated with tumor tissues, whereas *Bacteroidetes* and *Fusobacteria* were significantly reduced in tumor tissues [79]. The abundance of 22 bacterial and seven fungal genera significantly differed between the tumor group and the control group [80]. The abundance of the fungal genus *Aspergillus* in tumor tissue was negatively correlated with bacteria such as *Actinomyces*, Prevotella, and *Streptococcus* but positively correlated with *Aggregatibacter* [80].

Significant evidence supports the notion that chronic hyperplastic candidiasis is correlated with the medical background of immune-related disorders, head and neck cancer, radiotherapy, and chemotherapy [82]. Radiotherapy after head and neck cancer helps the development of fungus within the oral microbiome; *Candida albicans* is the most prevalent fungal species, followed by *Candida glabrata*, *Pichia kudriavzevii*, *Candida parapsilosis*, and *Candida tropicalis* [83].

Shay et al. conducted a study in the United States, and found that individuals with head and neck squamous cell carcinoma have a different oral mycobiome and bacteriome in comparison with healthy individuals, reflected by the salivary samples [84]. This study identified three fungal phyla and eleven bacterial phyla, with *Ascomycota* and *Firmicutes* being the dominant species [84]. The composition of the oral mycobiome and bacteriome differed between the two groups; *Candida albicans* and *Rothia mucilaginosa* were abundant, and *Schizophyllum commune* was reduced in subjects with head and neck squamous cell carcinoma [84].

Another study from Sudanconfirmed the mycobiome’s alteration in subjects with head and neck squamous cell carcinoma [85]. *Candida*, *Malassezia*, *Saccharomyces*, *Aspergillus*, and *Cyberlindnera* were the most relatively abundant fungal genera found in the salivary microbiome [85]. Among these, *Candida* was associated with a poor prognosis of the disease, while *Malassezia* was associated with a favorable prognosis [85]. This information sheds light on the potential role of the fungal microbiome in the development and progression of head and neck squamous cell carcinoma.

Heng et al. suggested a group of signature species composed of bacteria and fungi, which are closely associated with the sequence health-premalignancy-carcinoma in oral squamous cell carcinoma [86]. The identified signature species were *Prevotella intermedia*, *Porphyromonas endodontalis*, *Acremonium exuviarum*, and *Aspergillus fumigatus*, which prospered in oral carcinoma [86]. Whereas species like *Streptococcus salivarius* subsp. *salivarius*, *Scapharcabroughtonii*, *Mortierellaechinula*, and *Morchella septimelata* were diminished [86].

The involvement of the mycobiome in the process of carcinogenesis remains ambiguous. The implicated mechanisms include alteration of the immune response, facilitation of oral biofilm formation, and the production of carcinogenic metabolites such as acetaldehyde and nitrosamine [87]. Additionally, the intricate interactions within microbial communities are known to enhance biofilm production, adherence of microbes to oral epithelial cells, modulation of the immune response, and amplification of microbial virulence.

An umbrella review determined that oral dysbiosis may manifest in adults diagnosed with oral squamous cell carcinoma [88]. Regarding the link between the mycobiome and oral cancer, research has primarily concentrated on the genus Candida, which has been identified as prevalent among individuals with oral squamous cell carcinoma [88].

## 8. System Diseases and Oral Mycobiome

It seems that there is a two-way relationship between systemic diseases and oral mycobiome.

It has been observed that fungi could acclimate to higher temperatures, thereby augmenting their potential to replicate within the human body, particularly in the case of a high basal temperature [89]. This ability increases their pathogenic potential, even in fungal species that were previously deemed non-pathogenic [89].

Recent research has uncovered that *Candida albicans* possess an impressive ability to adapt metabolically, utilizing a diverse array of nutrient sources and pH conditions [90]. This fungus is also capable of manipulating its surroundings to cause disease and avoid detection by the immune system [90]. Furthermore, *Candida* is increasing with advancing age, with evidence suggesting that age-related changes in the immune system may contribute to its proliferation [57,91]. According to a study conducted by Nishimaki et al. [57], oral *Candida* was associated with advancing age, untreated decayed teeth, prosthetic teeth, salivary pH, and a low red blood cell count [57,92].

Furthermore, a recent study showed that structural alterations of oral mycobiota are significantly linked with pancreatic ductal adenocarcinoma [13]. The research revealed that patients with adenocarcinoma had a higher fungal abundance and lower fungal diversity when compared to healthy controls [13]. In addition, the abundance of *Basidiomycota* and *Unclassifed_p_Ascomycota* has been identified as a potential risk factor for adenocarcinoma [13].

Although recent findings have associated the malignancy with alteration in the composition of mycobiota, a specific mycobiotic signature does not occur; the only common characteristic was dysbiosis [93].

Dysbiosis in the oral cavity, particularly at a low diversity, can be linked to disorders like leukemia and mucormycosis [94]. A study by Shelburne et al. concluded that dysbiosis in the oral cavity created a permissive environment that allowed invasive mucormycosis to occur [94]. They suggested that the bacteriome, mycobiome, and their interactions might contribute to the etiopathogenesis of infectious diseases [94].

Emerging research linking the oral mycobiome with certain systemic disorders underlines the intricate relationship between the body’s microbial communities and overall health. While these studies have begun to sketch the outlines of a possible connection, the complexity of the mycobiome and its interactions with the host body presents challenges in defining a clear-cut mycobiotic signature associated with specific systemic diseases.

## 9. Summary of the Oral Mycobiome Role in Oral and Systemic Pathology

Summarizing the role of the oral mycobiome in the etiology of oral and systemic diseases, it is essential to approach the statement that dysbiosis of the oral mycobiome initiates the disease. 

The fungal species incriminated in oral and systemic pathology are highlighted in Table 2, while in Table 3 are enumerated species which demonstrated a negative correlation or protective factor with certain diseases.

Certain commensal fungal genera are associated with oral or systemic conditions. The genus *Candida*, particularly *Candida albicans*, is well known as a prominent oral fungus and is associated with different pathology. Its ability to transform from a commensal organism to a pathogenic is a key point in oral health. However, it is a significant member of the core mycobiome. *Cladosporium*, *Alternaria,* and *Fusarium* have not been linked to any specific pathology. This highlights the possibility that not all commensal fungi play a direct role in disease processes, or their roles are not yet fully understood. *Aspergillus* has a dual role; it is associated with negative outcomes like peri-implantitis and oral squamous cell carcinoma, indicating its potential pathogenicity under certain conditions. Interestingly, it also appears to have a protective effect against early childhood caries. This dual nature suggests that the impact of *Aspergillus* on oral health is context-dependent, possibly influenced by the abundance of specific species, host immune status or interactions with other microorganisms. *Aureobasidium* and *Rhodotorula* are considered protective factors for early childhood caries. Other genera and species were associated with disease, but they are not part of the core mycobiome. 

This review emphasizes the need for further research to understand the mycobiome’s role in health and disease. The new genomic technologies shed light on the composition of the oral mycobiome, but the complex composition of the mycobiome and its relationship with the bacteriome are not yet fully identified and understood. 

## 10. Suggestions for Future Research

Understanding the core mycobiome is vital for health and requires more research. This includes the identification of commensal species, studying the interactions between fungal species, their interactions with the host, and their relationships with other components of the microbiome.

In the future, research in oral microbiology should focus efforts primarily toward identifying the subtle mechanisms underlying general changes in the oral microbiome and the intimate processes by which individual genetic polymorphism interferes with general and oral health.

In addition, in the current era of genomics and proteomics, better knowledge of the host genetic variant that predisposes to both microbial colonization and the development of disease progression could contribute to a better understanding of oral disease pathogenesis and case management.

## 11. Limitation of the Study

The current research is subject to limitations. In the case of this study, designed as a narrative review, the main objective was to present the current state of scientific research in this field without focusing on methods of data selection and analysis from the literature. 

Limitations associated with this type of study could be addressed, clarified, as well as extended in future research. Thus, the results of this study should be interpreted with caution.

## 12. Conclusions

Infectious oral pathology involves a much more diverse fungal microbiota than previously thought. According to recent data, it is not only the complex result of the action of individual fungal pathogens but also the synergy driven between multiple fungal species and genera and dysbiosis which disrupts the ecologically balanced biofilm associated with oral homeostasis.

A better understanding of the genetic mechanisms underlying interactions between the host and exogenous or symbiotic fungal communities may provide promising avenues for further exploration of the oral mycobiome, which could help us better understand and monitor host response to existing antifungal therapies, as well as to identify promising new treatments.

## Figures and Tables

**Table 1:** The identified oral fungi in healthy adults.

Study	Ghannoum et al. 2010 [21]	Dupuy et al. 2014 [22]	Monteiro-da-Silva et al. 2014 [23]	Cheung et al. 2022 [24]
Samples	Oral rinse	Saliva samples	Oral rinse	Oral rinse
Method	DNA extraction and PCR analysis	Internal Transcribed Spacer	Internal Transcribed Spacer	Internal Transcribed Spacer
Fungal genera	*Candida* *Cladosporium* *Aureobasidium* *Saccharomycetales* *Aspergillus* *Fusarium* *Cryptococcus* Alternaria *Dothioraceae* *Eurotium* *Glomus* *Saccharomyces* *Teratosphaeria*	*Candida* *Cladosporium* *Alternaria* *Aspergilllus* *Fusarium* *Cryptococcus* *Aureobasidium* *Malassezia* *Irpex* *Cytospora/Valsa* *Lenzites/Trametes* *Sporobolomyces/Sporidiobolus*	*Candida* Rhodotorula *Penicillium* Aspergillus Cladosporium Trichoderma Scedosporium Alternaria Rhizopus	*Candida* *Fusarium* *Rhodotorula* *Alternaria* *Cladosporium*

**Table 2:** The summative table with oral fungal species implicated in different pathologies.

Fungal Species	Caries			Periodontitis	Peri-Implantitis	Oral Mycosis	Oral Fungal Malignancy and Premalignancy			Systemic Disease		
	Early Childhood Caries	Dentine Caries	Root Caries				Oral Lichenus Planus	Leukoplakia	Oral Squamous Cell Carcinoma	Anemia	Leukemia	Pancreatic Ductal Adenocarcinoma
*Acremonium exuviarum*									Heng et al. [86] 2022			
*Ascomycota*									Shay et al. [84] 2020			
*Aspergillus*									Mohamed et al. [85] 2021			
*Aspergillus fumigatus*									Heng et al. [86] 2022			
*Aspergillus restrictus*					Enghiad et al. [48] 2022							
*Basidiomycota*												Wei et al. [13] 2022
*Candida* genus				Urzua et al. [36] 2008 Canabarro et al. [37] 2013					Shay et al. [84] 2020 Mohamed et al. [85] 2021	Nishimaki et al. [57] 2019		
*Candida albicans*	Thomas et al. [27] 2016 de Carvalho et al. [30] 2006 Raja et al. [31] 2010 Xiao et al. [32] 2010 O’Connell et al. [33] 2020				Lafuente-Ibáñez de Mendoza et al. [47] 2021	Lu et al. [54] 2021 Imabayashi et al. [45] 2016	Zeng et al. [68] 2009	Gupta et al. [70] 2019				
* Candida dubliniensis *	O’Connell et al. [33] 2020	O’Connell et al. [33] 2020			Lafuente-Ibáñez de Mendoza et al. [47] 2021	Imabayashi et al. [55] 2016	Molkenthin et al. [42] 2022					
*Candida glabrata*						Lu et al. [54] 2021	Zeng et al. [68] 2009 Molkenthin et al. [42] 2022					
*Candida krusei*						Lu et al. [54] 2021	Zeng et al. [68] 2009 Molkenthin et al. [42] 2022					
*Candida parapsilosis*					Lafuente-Ibáñez de Mendoza et al. [47] 2021 Enghiad et al. [48] 2022	Lu et al. [54] 2021	Molkenthin et al. [42] 2022					
*Candida tropicalis*					Lafuente-Ibáñez de Mendoza et al. [47] 2021	Lu et al. [54] 2021 Imabayashi et al. [55] 2016						
*Cyberlindnera*									Mohamed et al. [85] 2021			
*Malassezia*									Mohamed et al. [85] 2021			
* Microdochium *	O’Connell et al. [33] 2020											
* Nigrospora oryzae *	O’Connell et al. [33] 2020											
*Saccharomyces*									Mohamed et al. [85] 2021			
*Unclassifed_p_Ascomycota*												Wei et al. [13] 2022
Dysbiosis									Mukherjee et al. [80] 2017 Shay et al. [84] 2020 Mohamed et al. [85] 2021		Shelburne et al. [94] 2015	Wei et al. [13] 2022

**Table 3:** The summative table with oral fungal species with negative correlation or protective factor with different pathologies.

Fungal Species	Caries			Periodontitis	Peri-Implantitis	Oral Mycosis	Oral Fungal Malignancy and Premalignancy			Systemic Disease		
	Early Childhood Caries	Dentine Caries	Root Caries				Oral Lichenus Planus	Leukoplakia	Oral Squamous Cell Carcinoma	Anemia	Leukemia	Pancreatic ductal Adenocarcinoma
* Aspergillus *	O’Connell et al. [33] 2020											
* Aureobasidium *	O’Connell et al. [33] 2020											
* Debaryomyces *	O’Connell et al. [33] 2020											
* Rhodotorula *	O’Connell et al. [33] 2020											
*Glomeromycota*									Mukherjee et al. [80] 2017			
*Morchella septimelata*									Heng et al. [86] 2022			
*Mortierella echinula*									Heng et al. [86] 2022			
*Schizophyllum commun*									Shay et al. [84] 2020			

## Data Availability

The data presented in this study are available upon request from the corresponding author.
